# Children are sensitive to the number of sources when relying on gossip

**DOI:** 10.1098/rsos.230375

**Published:** 2024-05-15

**Authors:** Asami Shinohara, Yasuhiro Kanakogi, Yuko Okumura, Tessei Kobayashi

**Affiliations:** ^1^ NTT Communication Science Laboratories, 2-4, Hikaridai, Seika-cho, Soraku-gun, Kyoto 619-0237, Japan; ^2^ Graduate School of Human Sciences, Osaka University, Osaka, Japan

**Keywords:** children, gossip, reputation, number of sources, selective learning

## Abstract

Gossip allows children to effectively identify cooperative or trustworthy partners. However, the risk of being deceived must be faced because gossip may be false. One clue for determining gossip's veracity is the number of its sources since multiple informants spreading identical reputational information about others might imply that another's moral traits are viewed unanimously among members of a social group. We investigated whether 7-year-olds (*N* = 108) would trust gossip from multiple independent sources. In our study, they received multiple pieces of positive/negative reputational information about one agent and neutral information about another agent by gossip from either single or multiple informants. Then they allocated rewards to and chose rewards from the gossip targets. The 7-year-olds acted upon positive gossip from multiple informants and did not rely on positive gossip from a single informant. By contrast, they relied on negative gossip regardless of the number of informants. In either valence, however, they were more likely to allocate rewards based on gossip from multiple informants than a single informant. This result indicates they are sensitive to an objective index, specifically the number of sources, for judging the veracity of gossip.

## 1. Introduction

Gossip is a ubiquitous phenomenon in human society. It serves as testimony about a social agent, defined as sharing reputational information about an absent third party [1,2]. Thanks to gossip, individuals can effectively acquire information about who is good or bad in their social group, especially when it is impossible to interact first-hand with others or make direct observations of their behaviour [3]. Unlike other types of testimony (e.g. testimony about the physical world [4]), a unique feature of gossip is that there can be social consequences for the people who are involved in it [5]. One potential social consequence is that individuals who receive gossip may decide how to interact with the subject of the gossip based on its content [3,6], potentially leading to the formation or alteration of cooperative relationships between them.

Not only adults but also children rely on gossip when choosing whom to cooperate with or believe. One study found that 5- and 7-year-olds allocated resources with targets of gossip based on the positive and negative gossip about the targets [7]. Seven-year-olds allocated resources to an agent based on gossip only when it contained negative information about the agent; 5-year-olds did not act on any reputational information. Shinohara *et al*. [7] also found that 7-year-olds rated the goodness of others based on positive and negative reputational information, but 5-year-olds only relied on negative information when evaluating others. Another study revealed that 5- and 7-year-olds relied on gossip to determine whom to believe [8]. In their study, children in pairs needed to decide the diet of a novel creature. Each child received conflicting information from a different informant, where one informant's reputation based on gossip was considered honest while the other's was considered dishonest. Then the child dyad was asked to choose an answer. They accepted information from an informant whose honesty (i.e. good reputation) was conveyed by gossip, but they did not accept information from an informant whose dishonesty (i.e. bad reputation) was disclosed by gossip. Consequently, the children not only based their behaviour on gossip in the manner of adults [3] but also paid attention to information through gossip when deciding whom to trust for correct answers.

Gossip is influential information even for children; however, individuals must exercise caution when trusting the information imparted by gossip because it can be manipulated or biased [9]. Some are eager to share false or exaggerated gossip [10,11]. For instance, people tend to share positive gossip about a friend to improve their status, whereas they spread negative gossip about an enemy to damage their reputation [12]. Even children understand that gossip can be biased by the relationship between a target and a gossip spreader [13]. If individuals trust false gossip, not only those who act upon it but also those who are its targets are harmed [10,14]. Thus, individuals must gauge carefully whether reputational information from gossip is reliable to minimize the risk of being deceived.

Then what cues do children use to assess the reliability of gossip? One possible approach is inferring the gossip spreader's intention to share accurate information (i.e. benevolence) [13]. To infer the intention of a gossip spreader, it might be necessary to consider the relationship between the gossip spreader and the target since this relationship determines the spreader's motivation to share good or bad gossip [12,13]; however, it is unlikely that children can always possess legitimate information about the relationships among people involved in gossip, especially when they are new to a social group. Also, confirming whether past gossip from a specific person is accurate may incur costs [6], making it difficult to determine whether the gossip spreader generally tends to spread gossip with benevolent intentions. In such situations, children need to rely on another cue unrelated to the gossiper's benevolence to judge the veracity of gossip; one potential cue is the number of independent sources of gossip [15]. Previous studies have found that multiple sources of gossip increase the gossip's veracity felt by adults [16,17]. The existence of multiple informants who are spreading the same kind of reputational information about others suggests that a strong consensus about another's moral traits is shared among multiple individuals [18,19]. Therefore, such a consensus might cause the children to judge that the gossip's content is true with some certainty, although it surely remains possible that incorrect information is spread by many sources. On the other hand, children may treat gossip from a single informant with some caution because this informant may bias the content of the gossip intentionally or unintentionally [11].

Indeed, even young children selectively trust information from multiple informants rather than a single informant when seeking a correct answer about a physical object [20–23]. For instance, 3- and 4-year-olds accept testimony about the name of a novel object from a majority (2 or 3 informants) rather than a single informant when the claims of the majority and the minority conflict [20]. Five- and six-year-olds endorse an opinion from multiple informants rather than a single informant, even when those informants do not even know the correct answer [21]. Children adopt the majority's opinion because it reflects an agreement among informants, thus rendering the information reliable. It is evident that the number of sources of information functions as a clue that suggests information reliability in the domain of selective learning about physical objects from testimony.

There is still a need to investigate whether children trust gossip from multiple sources. Gossip content, which can be either positive or negative information about an individual's behaviour or characteristics, differently influences children's social behaviour. For instance, 7-year-olds act upon a single instance of negative gossip but not positive gossip [7]. This characteristic leads to an important research issue: the impact of the number of gossip sources may depend on the gossip's valence. When children hear positive gossip, multiple sources (not just a single source) would function as a clue for its reliability. Positive gossip can lack sufficient influence to change children's behaviour [7]. Therefore, having multiple informants share positive gossip about one person will increase the reliability and intensity of the information, encouraging children to trust positive gossip. On the other hand, a previous study found that negative gossip impacts children's behaviour even when it is just one instance that comes from one source [7]. Hence, children would trust negative gossip even from a single source. However, the prediction that negative gossip from multiple informants is more reliable would not be undermined by considering the function of multiple sources [16,18]. Examining how multiple sources work depending on the valence of gossip will provide a broader picture of what kinds of information children weigh when they explore their social world.

The current study examined whether 7-year-old children would trust positive and negative reputational information through gossip from multiple independent sources. A previous study found that 7-year-olds can determine the veracity of gossip; they dismiss the gossip when its content is contradicted by what they actually see [24]. They also understand that the content of gossip is sometimes biased or manipulated and that its objective can be undermined [13]. Children not only understand this nature of gossip but also act upon it: Even children under 7 can refer to a number of sources when seeking correct information about an object's location or name [25,26]. These observations suggest that children of this age can determine whether gossip is valid by referring to the number of sources behind it.

**Figure 1.**
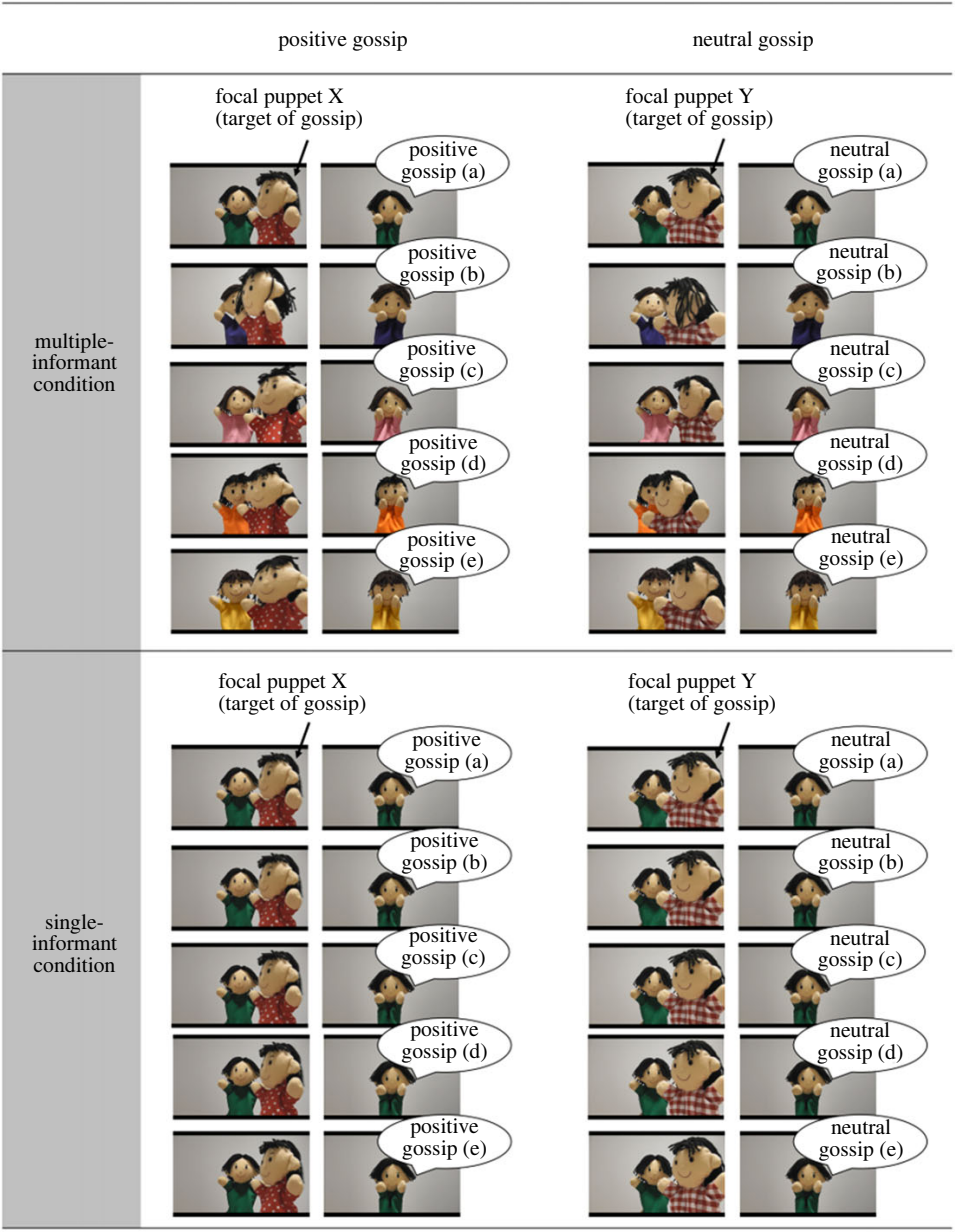
Examples of video stimuli in positive condition. In multiple-informant condition, children heard one positive gossip about focal puppet X from each of five informants (five pieces total) and one neutral gossip about focal puppet Y from each of five informants (five pieces total). In single-informant condition, they heard five pieces of positive gossip about focal puppet X from one informant and five pieces of neutral gossip about focal puppet Y from one informant. Children were assigned to either multiple-informant or single-informant condition. The presentation order of positive and neutral gossip was counterbalanced among participants. Positive gossip was replaced with negative gossip in negative condition.

This study employed a 2 (valence of gossip: positive/negative) by 2 (number of sources: multiple-informant/single-informant) between-subject design. The children heard gossip about two agents: reputational information about one agent (positive gossip in a positive condition and negative gossip in a negative condition) and neutral information about another agent (in both positive and negative conditions). The children were assigned to one of two conditions when learning gossip by video. In the multiple-informant condition (figure 1), they heard gossip with valence about one agent from multiple sources (i.e. five informants, each of whom shared one piece of gossip) and neutral gossip about another agent from multiple informants. In the second condition (single-informant condition; figure 1), the children heard five pieces of gossip with valence and five more pieces of neutral gossip from a single informant. We provided the same amount and the same gossip content in both single-informant and multiple-informant conditions to clarify that the number of sources (not the amount of gossip) mattered when the children trusted gossip. Thus, the only difference between the two conditions was the number of gossip sources. We used five sources to clarify the difference between single and multiple sources because a previous study found that 3- to 6-year-olds do not always perceive testimony from three or four sources as reliable when they are presented together with more repeated testimony from one source [27]. Although our study focused on an older age group (i.e. 7-year-olds), we used five sources because that seems to be the minimum number of sources required to function as a clue to gossip's reliability based on the results obtained with younger children.

After hearing the gossip, the children were shown two boxes containing many stickers or just a few (high reward or low reward) and asked to decide which gossip target should get which box (reward-allocation task) [7]. In addition, they were asked to choose from which of the two gossip targets they would like to receive a gift (reward-choice task) [7]. We adopted these reward tasks as indexes of the children's trust in gossip because such behaviors are related to a future cooperative relationship between an actor and a recipient of action [28,29]. If children decided who deserved a reward based on gossip, they would allocate a high reward to the agent linked to positive gossip and a low reward to the agent associated with negative gossip. If children determined who would confer benefit to themselves based on gossip, they would choose a gift from an agent with positive gossip but not from one with negative gossip. The children also rated the goodness of each gossip target on a 5-point Likert scale (−2: very bad to 2: very good).

As discussed above, the impact of the number of sources would differ depending on the valence of gossip. We predicted that 7-year-olds would act upon positive gossip from multiple informants, not just a single informant, whereas they would trust negative gossip regardless of the number of its sources. Overall, however, gossip from multiple informants would be more influential on children's behaviour than that from a single informant regardless of the valence of the gossip.

## 2. Method

This study was not pre-registered. All materials and the data of this study are accessible from the Open Science Framework (https://osf.io/sr8x6/) [30].

### 2.1. Participants

One hundred and eight 7-year-olds (*M* = 7.90 years, SD = 0.56; 57 boys), most of whom came from middle-class Japanese families living in small cities, participated in this study. The sample size was estimated by an *a priori* power analysis for a binomial test and a generalized linear model, where power = 0.80, alpha = 0.05, and with a medium effect size (*d* = 0.5 for the binomial test and OR = 2.47 for GLM) [27]. A power analysis revealed that *n* = 27 at minimum in each condition was necessary to achieve power = 0.80 in both statistical tests. The children were assigned to one of four conditions (*n* = 27 per condition) that varied in terms of the valence of gossip (positive or negative) and the number of sources (multiple or single informants): the positive/multiple-informant condition (15 boys and 12 girls: *M* = 7.93 years, SD = 0.67), the positive/single-informant condition (16 boys and 11 girls: *M* = 7.93 years, SD = 0.56), the negative/multiple-informant condition (13 boys and 14 girls: *M* = 7.84 years, SD = 0.42), and the negative/single-informant condition (13 boys and 14 girls: *M* = 7.91 years, SD = 0.59). Two additional children were excluded from the final analyses due to an experimental error (*n* = 1) and a failure to understand the task (*n* = 1). All of the participants were recruited from a database of parents who agreed to participate in children's studies, and they all provided written informed consent before the experiment. All consent and experimental procedures were approved by NTT Communication Science Laboratories (Study Number H26-002). The methods were carried out in accordance with relevant guidelines and regulations.

### 2.2. Materials

We played all of the videos on a 13-inch laptop (see Video stimuli below for details). Four boxes (10 × 10 × 3.5 cm^3^) were used for the reward-allocation and reward-choice tasks. We used seven puppets: two focal and five informant puppets. The focal puppets wore either a checked or polka-dotted shirt for easy discrimination. The informant puppets wore a green, blue, yellow, orange or pink shirt and spread gossip. We used photos of two focal puppets as a reference for the children.

### 2.3. Video stimuli

While watching videos, the children heard gossip about two focal puppets. They heard positive gossip about one focal puppet and neutral gossip about the other focal puppet in the positive conditions or, on the other hand, negative gossip about one focal puppet and neutral gossip about the other focal puppet in the negative conditions. These videos were created with reference to previous work [7,31]. Each contained a scene in which the informant puppet(s) shared gossip about the two focal puppets (figure 1). However, the number of informant puppets in the video differed between the multiple-informant and single-informant conditions. The videos began identically in all conditions. A focal puppet appeared wearing either a checked or polka-dotted shirt (counterbalanced across the gossip with valence and neutral gossip). Then the screen switched to an informant puppet in the centre of the screen. Next, the focal puppet passed from left to right in front of the informant puppet. After the focal puppet left the screen, the informant puppet provided gossip with valence (either positive or negative) or neutral gossip about that focal puppet. This scene was repeated five times, and the gossip content changed each time (table 1). Additionally, the number of informants varied by condition. In the multiple-informant condition, the children heard five pieces of positive or negative gossip about one focal puppet, and neutral gossip about the other one, from each of the five informant puppets. That is, each of the five informants provided one instance of gossip. The children in the single-informant condition also heard five items of positive/negative gossip and five items of neutral gossip from one informant. In the two informant conditions, the amount and content of the gossip were identical within the positive or negative conditions, but the number of sources differed. The presentation orders of the video content (i.e. positive/negative or neutral gossip) and the role of each focal puppet were counterbalanced across the children.

**Table 1.** Gossip content presented in videos.

positive gossip	negative gossip	neutral gossip
(a) helping a peer pick up a toy	(a) breaking a peer's toy	(a) playing in a sandbox
(b) sharing a toy with a peer	(b) stealing a toy from a peer	(b) taking a walk
(c) sharing treats with a peer	(c) stealing treats from a peer	(d) playing with a stuffed toy
(d) helping a peer clean a room	(d) hitting a peer	(d) playing on a swing
(e) helping a peer in trouble	(e) excluding a peer socially	(e) drawing a picture

### 2.4. Procedure

**Figure 2.**
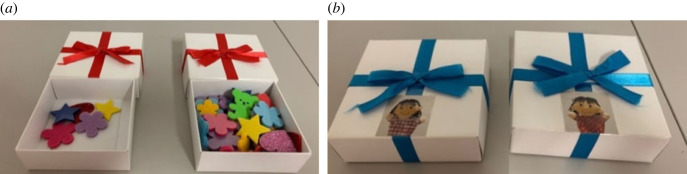
Boxes used for (*a*) reward-allocation and (*b*) reward-choice tasks. Box on left in reward-allocation task is a low reward, and box on right is a high reward. We attached a photo of one focal puppet to one box with a blue ribbon and a photo of the other focal puppet to the other box used in reward-choice task to easily discriminate which gift comes from which focal puppet.

All of the children were first taken to the experimental room and told that they were going to play games with boxes filled with stickers with two friends (i.e. puppets). The experimenter introduced the two focal puppets by showing photos of them. Then the children watched one set of gossip videos (i.e. either one of four conditions: positive/multiple-informant, positive/single-informant, negative/multiple-informant, or negative/single-informant). After watching the videos, they completed two reward tasks (i.e. reward-allocation and reward-choice) whose orders were counterbalanced across the children. The experimenter first showed them two boxes with red ribbons and two boxes with blue ribbons (figure 2). The former was for the reward-allocation task, and the latter was for the reward-choice task. To clarify which puppet the gift was from in the reward-choice task, one box with a blue ribbon had a photo of one focal puppet, and the other box had a photo of the other focal puppet. After the boxes were explained, the children were next told they were going to play a few games. In the reward-allocation task, the experimenter differentiated the gift quantity by showing that the two boxes with red ribbons contained either 40 stickers (high reward) or five stickers (low reward). The children decided which focal puppet should get which present and set that box in front of the puppet chosen to receive it. As the participants did this, the experimenter looked away to avoid biasing their decisions. In the reward-choice task, the experimenter explicitly explained that one box with a blue ribbon was a gift from a focal puppet and the other box with a blue ribbon was from the other focal puppet. Then the children indicated the box they wanted (first choice). The box not chosen by the children was coded as their second choice. All of the boxes contained two stickers, and the experimenter did not allow the children to look into or touch the boxes to prevent them from guessing the content. The children received their first choice and were allowed to open it after all experiments were finished. Finally, we explicitly asked the children to rate the goodness of each puppet on a 5-point Likert scale: three stars (2: very good), one star (1: good), a square (0: neither), one x-mark (−1: bad), and three x-marks (−2: very bad).

### 2.5. Coding

For the analysis, we coded the children's decisions as 1 if they acted on the gossip content in each reward task, and 0 otherwise. A score of 1 was assigned when a child exhibited the following behaviours: (1) allocating a high reward to the puppet with positive gossip in the reward-allocation task, (2) choosing a gift as a first choice from the puppet with positive gossip in the reward-choice task, (3) allocating a low reward to the puppet with negative gossip in the reward-allocation task, and (4) not choosing a gift as a first choice from the puppet with negative gossip in the reward-choice task.

### 2.6. Approach to analysis

We performed binomial tests and used a generalized linear model with binomial error distribution (GLM) in our primary analyses. GLMs were applied in each reward task.^1^ We calculated bootstrapped 95% confidence intervals (*R* = 2000) for the primary analyses. We also calculated the odds ratio in GLM.

## 3. Results

All of the statistical analyses were conducted with R statistical software (Version 4.0.2 [32]).

We first confirmed that each participant's choices (0/1) in the reward-allocation and reward-choice tasks were highly correlated (*r* = 0.71) in the positive and negative conditions. Overall, 99 of 107 children made the same choice in both tasks.

### 3.1. Reward-allocation task

**Figure 3.**
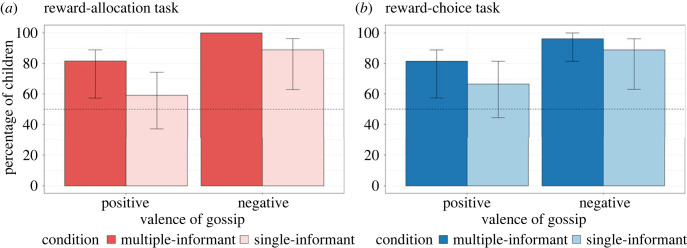
Percentage of children who acted upon gossip in (*a*) reward-allocation and (*b*) reward-choice tasks. Dashed lines indicate chance level (50%). Error bars indicate bootstrapped 95% confidence intervals. Since the data have no variance in reward-allocation task in negative/multiple-informant condition, we were not able to compute bootstrapped 95% confidence intervals for them.

First, we conducted a binomial test for each condition (positive/multiple-informant, positive/single-informant, negative/multiple-informant and negative/single-informant) to determine whether the number of children who allocated rewards based on gossip differed significantly from chance level (50%). Our analysis found that the number of children who allocated rewards based on positive gossip was not significantly different from chance in the single-informant condition (figure 3*a*, 16 of 27, *p* = 0.442). In the multiple-informant condition, they were likely to allocate rewards based on positive gossip (figure 3*a*, 22 of 27, *p* = 0.002). Only in the multiple-informant condition did children tend to allocate a high reward to the puppet with positive gossip in the reward-allocation task. In the negative conditions, we found that the number of children who allocated rewards based on negative gossip was significantly higher than chance, in both the single-informant (figure 3*a*, 24 of 27, *p* < 0.001) and multiple-informant conditions (figure 3*a*, 27 of 27, *p* < 0.001). Most children, in both conditions, allocated a low reward to the puppet with negative gossip in the reward-allocation task.

Second, we used a binomial GLM to analyse whether the children's propensity to allocate rewards based on gossip would differ depending on the number of sources (multiple-informant or single-informant condition) and the valence of gossip (positive or negative condition). The outcome variable was children's decisions in the reward tasks (0/1). Initially, we tested the significance of a two-way interaction between the number of sources and the valence of gossip; however, the interaction was not significant (LRT = 1.65, *p* = 0.200). Therefore, we dropped the two-way interaction in the final model; the final model only included the main effects of the number of sources and the valence of gossip. The final model revealed a significant effect of the number of sources (LRT = 5.95, *p* = 0.015). The children were more likely to allocate rewards based on gossip in the multiple-informant condition than in the single-informant condition (OR = 3.94, 95% CI [0.07, 1.31]). Furthermore, a significant effect was revealed for the valence of gossip (LRT = 12.27, *p* < 0.001). The children were more likely to act upon negative than positive gossip (OR = 7.92, 95% CI [0.28, 1.78]).

### 3.2. Reward-choice task

All statistical approaches were identical to those for the reward-allocation task. A binomial test revealed that the number of children in the positive condition was not significantly different from chance level in the single-informant condition (figure 3*b*, 18 of 27, *p* = 0.122). On the other hand, the children were more likely to choose the rewards based on positive gossip in the multiple-informant condition (figure 3*b*, 22 of 27, *p* = 0.002). Only children in the multiple-informant condition tended to choose a gift from the puppet with positive gossip. In the negative conditions, we found that the number of children who chose a gift based on negative gossip was significantly higher than chance in both the single-informant (figure 3*b*, 24 of 27, *p* < 0.001) and multiple-informant conditions (figure 3*b*, 26 of 27, *p* < 0.001). Most children, in both conditions, did not choose a gift from a puppet with negative gossip.

We also used a GLM with a binomial distribution to compare the children's propensity to act upon gossip between the number of sources and the valence of gossip. A two-way interaction between the number of sources and the valence of gossip was not significant (LRT = 0.086, *p* = 0.770); therefore, we dropped the interaction in the final model, which only included the main effects of the number of sources and the valence of gossip. The final model revealed the significant effect of the valence of gossip (LRT = 7.16, *p* = 0.007). Here, the children were more likely to act upon negative than positive gossip (OR = 4.53, 95% CI [0.11, 1.47]). On the other hand, the main effect of the number of sources did not reach significance (LRT = 2.60, *p* = 0.107), even though the children tended to act upon gossip from multiple informants more than from a single informant (OR = 2.41, 95% CI [−0.20, 1.05]).

### 3.3. Goodness rating

**Figure 4.**
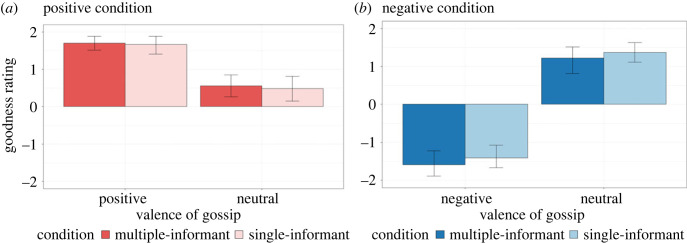
Mean of goodness ratings in (*a*) positive and (*b*) negative conditions. Error bars indicate bootstrapped 95% confidence intervals.

Table 2 and figure 4 show the results of the goodness rating. We ran a separate two-way ANOVA on the children's ratings in the positive and negative conditions using the number of sources (multiple informants or a single informant) as a between-subject variable and the valence of gossip (positive/neutral or negative/neutral) as a within-subject variable. In the positive condition, we found a significant main effect of the valence of gossip (*F*_1,36_ = 62.16, *p* < 0.001, *η*^2^ = 0.39) but not for the number of sources (*F*_1,52_ = 0.16, *p* = 0.69, *η*^2^ = 0.00) or the interaction effect (*F*_1,52_ = 0.02, *p* = 0.90, *η*^2^ = 0.00). The children rated a puppet whose positive gossip was shared (*M* = 1.69, SD = 0.58) more positively than a puppet whose neutral gossip was shared (*M* = 0.52, SD = 0.86). In the negative condition, we found a significant main effect of the valence of gossip (*F*_1,52_ = 211.126, *p* < 0.001, *η*^2^ = 0.73). However, we found no significant main effect of the number of sources (*F*_1,52_ = 2.21, *p* = 0.14, *η*^2^ = 0.00) or interaction effect (*F*_1,52_ = 0.01, *p* = 0.93, *η*^2^ = 0.00). The children rated a puppet whose negative gossip was shared (*M* = −1.5, SD = 0.88) more negatively than a puppet whose neutral gossip was shared (*M* = 1.30, SD = 0.84).

**Table 2.** Mean (SD) rating scores in each condition.

condition	rating score	
	positive gossip	neutral gossip
multiple-informant condition	1.70 (0.47)	0.56 (0.80)
single-informant condition	1.67 (0.68)	0.48 (0.94)
	negative gossip	neutral gossip
multiple-informant condition	−1.59 (0.89)	1.22 (0.97)
single-informant condition	−1.41 (0.89)	1.37 (0.69)

## 4. Discussion

This study aimed to investigate whether 7-year-olds trust positive and negative gossip from multiple independent informants. The children acted upon positive gossip from multiple informants but not from a single informant. Conversely, they relied on negative gossip regardless of the number of sources. We also compared the impact of gossip from multiple and single informants on children's behaviour. In tasks where children allocated a reward, they were more likely to trust both positive and negative gossip from multiple informants than from a single informant. Additionally, a similar trend was observed when children chose a reward based on gossip, although the *p*-value did not reach significance. Notably, we found that children trusted negative gossip more than positive gossip in both reward tasks. Our findings suggest that children judge the veracity of gossip based on the number of sources, even when exposed to the same amount of gossip in both the multiple-informant and single-informant conditions.

We posit two possible reasons why the children were more likely to act upon gossip from multiple sources than from a single source when allocating rewards. First, multiple sources functioned as an index of the likelihood that one's moral traits adhere to consensus views held by multiple people, which encouraged the children to trust the reputational information from gossip. Even if a single informant repeatedly shares the same kind of gossip content, its objectivity does not sufficiently increase because it might reflect the informant's bias. Thus, children might still be at risk of being misled when hearing multiple pieces of gossip from a single source. On the other hand, having multiple informants, who spread the same kind of information about a person's moral traits, functions as a more objective clue to determining whether gossip, including that about a person's reputational information, can be regarded as credible information shared among a social group [18]. This possible consensus behind multiple informants, which increases the gossip's reliability, might motivate children to rely on gossip from multiple sources rather than a single source. The second reason might be that they tend to accept the majority's opinion [33]. Previous studies show that children tend to conform to the majority, for example, when learning a particular action from the majority, even abandoning their behavioural preference to solve a problem [34], or when following the majority's decision in perceptual judgement tasks, even if they know that the answer is wrong [35,36]. As one underlying psychological motivation to conform to the majority, individuals deal with various social drives, including the desire to be affiliated with the majority [37]. The children in our study were possibly motivated to be accepted by the majority, so they were more likely to act on gossip from multiple sources than from a single source. Further studies are needed to deepen our understanding of why children rely on gossip from multiple sources.

However, we did not find a significant difference in children's reward choices based on gossip between instances when gossip was spread by multiple sources and by a single source, despite observing a trend indicating that children were more influenced by gossip from multiple sources. Although the children's reward allocation was influenced by the number of gossip sources, we must acknowledge that the effect of the number of sources on the children's reward choice was insufficiently strong. This minor effect may be attributed to the children's desire to avoid missing out on the possibility of receiving a high reward (i.e. avoiding the risk of loss), which led some children to trust gossip even from a single source. On the other hand, we found that the children's choices of the reward-allocation and reward-choice tasks were highly correlated, suggesting that these two tasks were related. Hence, we remain sceptical that what was measured in the two tasks can be treated as different components. Further examination is necessary to explore how the type of children's behaviour is affected by the number of gossip sources.

Children's tendency to rely on gossip differed depending on the valence of gossip. The children acted upon positive gossip only from multiple sources, although they trusted negative gossip from both single and multiple informants. This result's explanation may be related to the risk of trusting false gossip. The risk of being deceived by positive gossip is relatively high when such positive reputational information about others is false and when those described by it are actually malevolent. Children who trust such false positive gossip may eagerly interact with such a person [38]. However, at the same time, they might be exploited or harmed by a person who is actually ill-intentioned. On the other hand, one possible risk of trusting false negative gossip is that the children might miss the potential benefits of interacting with the gossip's target. Consequently, a more adaptive strategy is to trust negative gossip and avoid the risk of being threatened or exploited (e.g. [39]), even if such negative gossip is inaccurate. Indeed, humans are more likely to avoid losses than to pursue profits [40]. Compared with the risk of trusting false negative gossip, the risk of trusting false positive gossip might threaten their welfare or even their lives. This potential risk of trusting false positive gossip may make children more sensitive to the number of gossip sources: in other words, the extent of the positive gossip's veracity. It is also conceivable that the children were more influenced by negative gossip than positive gossip because the impact of the valence of information on their behaviour is different between these two types [7]. It may be functionally adaptive for children to adjust their behaviour based on negative gossip simply to avoid harmful situations caused by future interactions with a malevolent person [39,41]. Negative gossip may also elicit physiological arousal, which might cause children to trust and act upon it [42,43]. Due to such a human tendency, the children's behaviour was determined based on negative gossip from just one source. Compared to negative gossip, positive gossip might be a weaker, less meaningful signal of others' prosocial traits lacking any impact on children's behaviour [41]. Positive gossip may become powerful only when more than one informant conveys an identical kind of gossip, which motivates children to act upon it.

We also found evidence that the children rated the goodness of others based on the content of the positive and negative gossip from both multiple and single sources. This result is consistent with a previous study [7]: 7-year-olds do not form their attitudes based on one instance of positive reputational information from gossip, whereas they rate the goodness of agents based on such positive information, and their attitudes and goodness rating of others are influenced by negative gossip. Rating the goodness of agents based on gossip assumes that 7-year-olds can understand and process the content of such gossip. This assumption negates the following possibility: children who heard reputational information from a single informant failed to understand and process its content, which led to their weaker tendency to act upon gossip from a single informant than when it was from multiple informants. Although 7-year-olds can process reputation information from gossip regardless of the number of sources (i.e. multiple or single), they might be more likely to accept multiple sources as having greater credibility when deciding how to behave toward a target of gossip.

Although our results suggest that children are sensitive to the number of sources when acting on gossip, this might have been caused by the differences in their attention to the videos between the two conditions. Multiple informants appeared in the video of the multiple-informant condition, while only one appeared in the video of the single-informant condition. More informants in the multiple-informant condition probably grabbed more of the children's attention to the video stimuli, which might have contributed to better discrimination between the two puppets based on the gossip's content. If so, the children in the single-informant condition are less likely to discriminate between the two gossip targets. However, the results of our goodness rating confirmed that the children in both conditions discriminated between the two agents based on the gossip content, even at the end of the experiment. This finding eliminates the possibility that more informants attracted the children's attention and caused the differences between the number of sources. Therefore, perhaps our result cannot be explained by any attentional differences of the children between the two informant conditions.

An interesting future study would be to investigate how the interactions between the number of sources and such informant characteristics as benevolence, or the relationship with a gossip target, influence the children's faith in gossip. Since they understand that friendships between informants and gossip targets [13] affect the content of such information, examining how these effects change when multiple sources are involved might be fruitful. In addition, it is worth revealing how the mixed valance of gossip influences children's trust in it. When individuals learn about others through gossip, its content is often inconsistent: some people offer praise, while others make disparaging remarks [12]. When children hear conflicting gossip about the same person, how do they evaluate and behave toward him or her? If the amount of positive gossip exceeds the negative, which do they trust? How do they behave toward the gossip's target? Such questions must be answered to understand children's trust in gossip.

In conclusion, we found that 7-year-olds were more likely to rely on gossip from multiple sources than from a single source in deciding how to allocate rewards toward the target of gossip. This result suggests they are sensitive to an objective index (i.e. the number of gossip sources) when determining the gossip's credibility. Our finding also indicates that children may understand the inherent nature of gossip itself. Although its content may be biased in some ways, multiple sources of gossip increase its perceived veracity. Our results contribute to a better understanding of how children expand their social world by showing they selectively interact with others through gossip.

## Data Availability

The materials and the data that support the findings of this study are accessible from the Open Science Framework (https://osf.io/sr8x6/) [30].

## Footnotes

1 When performing a sample-size estimation, we did not include the type of task as a predictive variable. Nevertheless, the necessity to include the type of task as a predictive variable and implement a GLMM became apparent later; however, we used separate GLMs for each task because we lacked adequate statistical power to perform a direct comparison between the two reward tasks.
